# Home monitoring as a useful extension of modern tele-ophthalmology

**DOI:** 10.1038/s41433-020-0964-3

**Published:** 2020-05-13

**Authors:** Livia Faes, Lucas M. Bachmann, Dawn A. Sim

**Affiliations:** 1 Moorfields Eye Hospital NHS Foundation Trust and UCL Institute of Ophthalmology, London, UK; 2 Cantonal Hospital Lucerne, Lucerne, Switzerland; 3 Medigntion Inc, Zürich, Switzerland

**Keywords:** Health services, Retinal diseases

The self-measurement of clinically relevant parameters by the patient between two doctor consultations is an important pillar of modern medical care [1–3]. Several large programmes have been launched in recent decades under the term home measurement, which have subsequently shown clear effects in favour of the timely detection of a worsening of disease and its targeted prompt treatment [4–7]. In addition to these immediate clinical effects, home measurements also lead to patients gaining autonomy in the self-responsible treatment of their disease and—presumably through this commitment—showing a significantly higher adherence to treatment [6]. Home assessment programmes may also reduce emergency consultations and organizational inefficiencies in outpatient and inpatient care although evidence remains inconclusive [4].

Home measurements have been around for a long time and range from measuring body temperature during infection monitoring to regular body weight measurements in heart failure or chronic kidney disease. More recently, research in the fields of blood pressure measurement for hypertension, peek-flow measurement for chronic obstructive pulmonary disease, asthma and glucose measurement for diabetes has shown benefits of home monitoring [1, 5–7]. Using simple therapy schemes, patients can make treatment adjustments on their own if the home measurements indicate this.

In the field of ophthalmology, the Amsler grid is one of the most widely used home monitoring instruments [8]. The Amsler grid identifies metamorphopsia, perceived distortions of visual stimuli mainly due to a mechanically distorted retina. Such distortions are i.e. typical in (advanced) stages of age-related macular degeneration (AMD) due to drusen or intraretinal fluid, and in diabetic macular oedema [9–11]. In these diseases, management is often guided by data obtained from sporadic outpatient visits. The dynamic fluctuations in chronic eye diseases contain valuable data that we cannot capture. Home monitoring by patients measurements offers a novel quality of clinical data, and not only reduces their need for physician visits, but also enables the collection of high-quality, structured data in an intramural environment for personalized and targeted management.

Recently, several digital home measurement tests have come onto the market [12]. In recent years, preferential hyperacuity perimetry (PHP, Notal Vision Inc) has established itself in patient self-testing for AMD [13]. A clinical study showed a lower reduction in visual acuity compared with standard care using the PHP test [14]. The test runs on a standalone device connected to the company’s data centre via a wireless service. myVisiontrack® and Alleye are the only two FDA-approved medical software applications that run on mobile devices. myVisiontrack® uses a shape discrimination task [15]. The Alleye test has several similarities with myVisiontrack®, but examines a larger central visual field [16, 17].

The implementation of home measurement programmes alone can have a positive effect on medical care. Ideally, however, the home measurement systems are linked to the digitized processes of further care. As digitalization progresses, completely new integrated care systems emerge which are characterized by significantly higher efficiency [2]. Many experts believe that these modern approaches can meet the challenges posed by demographic changes and the associated increase in the number of patients. It is estimated that the proportion of people over 60 years of age will double by 2050 and that one in five people will be 60 years or older. The demand for ophthalmic services exceeds supply [18].

Although the number of ophthalmologists is increasing, the UK has one of the lowest ratios of ophthalmologists per capita in the developed world, and their number is growing only half as fast as the population over 60 years of age [19]. The challenge is to maintain timely and high-quality care as resources become scarcer. The impact of this imbalance between supply and demand is illustrated by the recently published figures of 20 patients per month facing severe vision loss and waiting for access to ophthalmic services [20].

Therefore, over the past two decades, an important foundation has been laid for the efficient implementation of integrated digital ophthalmic care [21, 22]. Currently, the store-and-forward model of tele-ophthalmology is in use, where images are taken at a different time and place than when assessed by a trained grader, best illustrated by the United Kingdom’s National Diabetic Eye Screening Programme (DESP) [23]. The DESP has improved access to care, with 76% of patients eligible for screening receiving annual retinal imaging. At the same time, risk populations of diabetic retinopathy will be screened to be identified for early treatment in hospital-based services. As a direct result of this programme, diabetic retinopathy was no longer the leading cause of blindness in England and Wales for the first time in five decades in 2009 and 2010 [24]. In return, the introduction of the screening programme led to an increase in referrals for further investigations, which in large numbers did not reveal any abnormal clinical findings, but which placed an additional burden on the institutions due to the increased number of patients.

The analysis of these mechanisms led to the concept of the “virtual clinic”, which aims to provide additional capacity for unmet needs within the National Health Service (NHS) [25]. Initial studies have shown that patients do not need personal interaction with a doctor every time they stay in hospital and that a secure, efficient service can be offered virtually. Virtual clinics have been tested in several subspecialties, including medical retinas, glaucoma and emergency ophthalmology, with a number of programmes developed over the last two decades. While it is clear that individual programmes have been successful, none has scaled to a national level.

Widespread digital health transformation in ophthalmology has been challenged by several factors. Clinicians have raised concerns regarding its security (i.e., quality of imaging, encryption), the patchy evidence base (i.e., medical smartphone apps), the dependence on a minimal level of technological infrastructure and the costs for the set up and maintenance of the service [26–28]. From a patient’s perspective, apprehensions in regards to usability and impeded relationships with health care professionals have been highlighted[27]. Indisputably, the complementary role of digital health care needs to be empathized at this point. Patients with complex needs (i.e., dementia or significant social deprivation) require face-to-face support. It is anticipated however, that there would be more time for clinicians to focus on patients in need for traditional consultations if routine ailments were being dealt with by digital care. An increasing number of systematic reviews assessing the role, impact and cost-effectiveness of telemedicine services showed the positive yield of them when applied in appropriate settings and involving an interdisciplinary team of clinicians, funders, governmental and non-governmental institutions [29–31].

Therefore—despite the current barriers—the future of tele-ophthalmology seems bright.

The expansion of telemedical services is the logical response to current and future supply bottlenecks in ophthalmology. Further development will take place in stages and will include data-specific, technological and political steps.

1. From the point of view of data integration, the inclusion and provision of all care-relevant data is crucial. The inclusion of data from patient home measurements is a decisive and essential component, because the density of relevant data increases significantly and allows additional insights into disease progressions, which are missing in classical data surveys. The inclusion of this data has two major effects: clinical and efficiency-enhancing. The increase in the involvement of the patient and his ability to participate in the treatment of his disease is supplemented by the improvement of the downstream telemedical care paths.
2. An integrated telemedical software solution is necessary for a comprehensive introduction because several parallel software programmes lead either to data silos or interface problems. The technology must enable the collection of structured clinical information and the bidirectional flow of this information between patient, community and hospital. If these infrastructure requirements are met, automated classification systems such as artificial intelligence algorithms can be used efficiently.
3. From a political point of view, a binding commitment is necessary. However, this seems to be the case since a recently published 10-year long-term plan of the NHS sets exactly these priorities. The plan aims at transforming services and overcoming the imbalance between supply and demand in health care. Digital technologies are described as a critical part to achieve this goal and to make care in the community as secure and possible as possible. An integrated telemedicine with patient home measurements maps these goals in an optimal way.
